# The Connection between Knowledge and the Nutritional Behaviour of Parents and the Occurrence of Overweight and Obesity among Preschool Children—A Pilot Study

**DOI:** 10.3390/nu16010174

**Published:** 2024-01-04

**Authors:** Aleksandra Mazurkiewicz, Ewa Raczkowska

**Affiliations:** Department of Human Nutrition, Faculty of Biotechnology and Food Science, Wrocław University of Environmental and Life Sciences, 37 Chełmońskiego Street, 51-630 Wrocław, Poland; 114911@student.upwr.edu.pl

**Keywords:** obesity, overweight, nutrition, nutritional behaviour, nutritional knowledge, preschool children

## Abstract

The phenomenon of overweight and obesity constitutes a threat for health and children’s lives at preschool age. Eating habits formed during this period seriously affect not only future dietary preferences but also the development of diet-related diseases. The purpose of the present study was the assessment of the relationship between the state of knowledge of children’s nutrition and parents’ eating behaviour and the prevalence of overweight and obesity in children aged 4–6 years. The study was conducted using 200 parent–child pairs. The behaviour and nutritional knowledge of parents was assessed using a questionnaire method. In contrast, anthropometric measurements were taken among the children. Nutritional disorders were noted in 46.5% of children of whom 39.0% struggled with overweight and 7.5% with obesity. The study showed that the children of parents with higher levels of nutritional knowledge were more likely to develop overweight and obesity. It also proved that irregularity of parental food intake predisposed the development of excessive body weight in children (*p* = 0.0049). Therefore, it is reasonable to undertake further investigation into factors implying the development of nutritional disorders among those youngest. Early recognition of dietary mistakes can contribute to their elimination at an early age.

## 1. Introduction

Overweight and obesity are described as an epidemic of the 21st century. In addition, they are classified as a civilisation disease whose development is closely correlated with technological progress [1].

The World Obesity Federation (WOF) and the World Health Organization (WHO) report that nearly 2.6 billion people globally are struggling with excessive body weight, including more than 1 billion with obesity. These organizations pay particular attention to the prevalence of the problem among the youngest part of the population—preschool children. In accordance with current statistics, approximately 39 million people included in this group suffer from obesity [2,3,4]. According to predictions, if the situation does not improve, the number of people suffering from obesity will rise to around 1.9 billion by 2035. It is worth highlighting the fact that excessive body weight will be increasingly recognized not only among adults but also among children. As stated by some experts, around 400 million children could face this problem in the next 12 years. Moreover, the WHO warns that around 167 million people will deal with health risks as a result of being overweight or obese [3,5,6].

It has been pointed out that the development of excessive body weight in the early stages of life is particularly dangerous. According to a 9-year observation, it was shown that children diagnosed as being overweight at the age of 5 years have a fourfold increased risk of obesity compared with control group members with a body weight in accordance with current norms [7]. What is more, the study by Llewellyn et al. (2016) demonstrated that high BMI values recognised among children later qualified for a diagnosis of diabetes in adulthood [8]. In addition, children who are burdened by overweight or obesity may have difficulty with concentration and attention as a result of poor mood and sustained tension. This may have a negative impact on academic performance and may exacerbate disorders of a psychological nature [9].

Due to the high prevalence of overweight and obesity and their health consequences, they pose a serious threat to society [10]. It is disturbing that excessive body weight is being diagnosed in increasingly younger people globally, in Europe and in Poland. The intensive development of excessive body weight is also closely linked with the COVID-19 pandemic, which ran from 2020 to 2022. Remote learning and working modes as well as reduced opportunities to leave the home environment translated into reduced levels of physical activity and a greater tendency to overeat. This was positively correlated with feelings of stress. It has been shown that the lockdown period contributed to an increase in consumption of all food groups relative to the pre-pandemic period. In accordance with available studies, the deterioration of eating habits can be referred to not only in adults but also in children and adolescents. Furthermore, researchers emphasise that, as a result of the deterioration of dietary habits and an accordingly inappropriate lifestyle, significant weight gain was observed in the youngest part of the population after the pandemic [11,12]. Therefore, it can be concluded that preschool children are particularly vulnerable to the occurrence of overweight and obesity [13]. This period is considered to be critical in the development of overweight due to children being at their greatest vulnerability to their surroundings. A key role during this time is played by parents since they are involved in shaping their children’s specific eating habits and preferences [14,15]. They are responsible for the correct supply of nutrients, variety and quantity of meals consumed by the child. Furthermore, they decide on the choice of specific foodstuffs and subsequently how these articles are prepared. It is therefore emphasised that inappropriate parental eating behaviour as well as their inadequate knowledge of the principles of healthy nutrition lead to weight gain in children because of abnormalities perpetuated over the years [16,17,18].

The low level of knowledge about the causes and consequences of excessive body weight leads to its misconception as a “temporary condition” [19]. It is worth noting that even in the medical field, and more specifically in the paediatric field, obesity and overweight are not recognised as separate disease entities and therefore children burdened by them are not usually qualified for medical intervention [20,21]. This view is inconsistent with the International Statistical Classification of Diseases and Health Problems (ICD-11) effective from 01 January 2022. It has been adopted by the WHO and defines obesity and overweight as diseases, assigning them the designations 5B81 and 5B80.0 [22].

Taking preventive measures and carrying out examinations in the indicated area is significant because it allows us to minimize the risk of later complications and thus to achieve a higher quality of life [23,24]. Therefore, the aim of this study was the assessment of the relationship between parental knowledge and dietary behaviour and the prevalence of overweight and obesity in preschool children. Moreover, the aim of the study was to appraise the prevalence of excessive body weight in the indicative age group. Research that has been conducted so far does not present an unidirectional view concerning the impact of parental nutritional knowledge on the maintenance of children’s correct nutritional status. According to some authors, a higher level of nutritional knowledge among parents may translate into a reduced risk of overweight and obesity among preschool children [25,26,27]. In contrast, other scientists suggest that the above-mentioned relationship does not exist and therefore further studies are needed [28,29,30]. Referring to the influence of parental dietary behaviour on the development of excessive body weight in children, it is possible to observe total consensus in the views presented by specialists. Authors unanimously admit that dietary mistakes perpetuated in the family sphere are closely associated with an increased risk of overweight and obesity’s development in minors [31,32,33,34,35,36,37,38]. In our own study, it was hypothesized that parents’ inadequate knowledge and their inappropriate eating behaviour are significantly associated with the prevalence of excessive body weight among preschool children.

## 2. Materials and Methods

### 2.1. Study Design and Sample

The study was conducted between October and December 2022 involving parent-child in preschool age (4–6 years) pairs. The children attended public kindergartens in the Province of Lower Silesia. The Lower Silesian Voivodship is located in the southwest of Poland and is one of the most dynamically developing areas of the country. It is also worth highlighting that this region, according to available data, is among the top in terms of average population density. Moreover, the Lower Silesian Voivodship shares borders with the Czech Republic and the Federal Republic of Germany, which is undoubtedly an asset in the context of its location [39]. Another factor determining the choice of the above-mentioned area for analysis was the possibility of carrying out research in the specific educational establishments. Therefore, this choice was based on subjective knowledge about the population regarding the preschool children’s age. The choice of Lower Silesia was also justified by the possibility of carrying out further nutritional education for parents and children in the area that is locally closer to the authors. 

**Figure 1:**
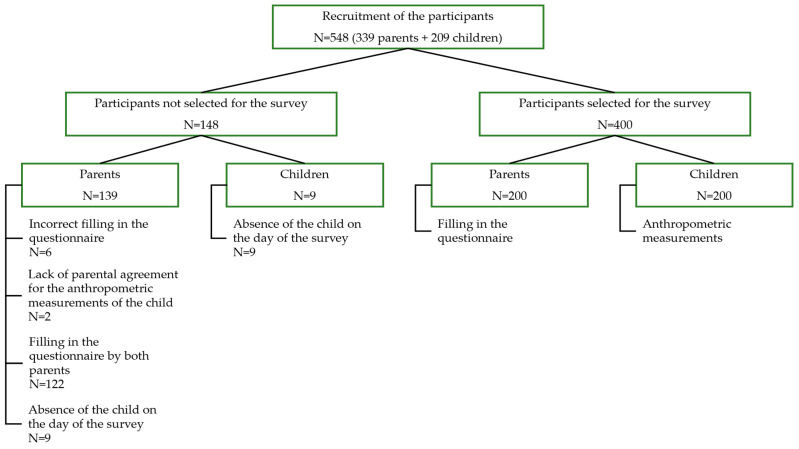
The general scheme of the conducted research.

The general scheme of the study is shown in Figure 1. The study involved 339 parents and 209 children aged 4–6 years. Eventually, 200 parent–child pairs were included in the statistical analyses. The largest part of the survey population was constituted by people who indicated a city of up to 100,000 inhabitants as their place of residence (*n* = 156; 78.0%). The small number of respondents lived in cities with more than 100,000 inhabitants (*n* = 18; 9.0%). The rest of the community (*n* = 26; 13.0%) were parents from rural areas. More than half (*n* = 107; 53.5%) of the respondents had a university degree. In both groups of girls’ and boys’ caregivers, a similar percentage of respondents declaring a higher level of education was registered at 26.5% and 27.0%, respectively. Slight differences between the indicated groups were also observed when it came to secondary education. In the group of girls’ caregivers, 15.5% of people selected this answer, and in the group of boys’ caregivers, 15.0% of people did so. The vast majority of parents (*n* = 171; 85.5%) described their professional status as working, including 42.0% (*n* = 84) of girls’ parents and 43.5% (*n* = 87) of boys’ parents. The least numerous groups were students (*n* = 1; 0.5%), annuitants (*n* = 2; 1.0%) and people describing another professional status (*n* = 6; 3.0%)—maternity leave, parental leave, running their own business and caring for a disabled person. The research was approved by the Research Ethics Committee of Wrocław University of Life Sciences (no. 14/2023 of 29 June 2023). The study was conducted in accordance with the guidelines of the Declaration of Helsinki [40]. In addition, informed written consent for the study was obtained from head teachers of kindergartens and children’s parents. The survey was anonymised.

### 2.2. Questionnaire

The survey was unsupervised and was also conducted without an interviewer. Therefore, questionnaires were given to both mothers and fathers. The research adopted a pairwise analysis of one parent–child. In a case where the questionnaire was completed by two parents, the mothers’ responses were included in the analysis. Indeed, it has been shown that mothers are largely responsible for the nutrition of their children [41]. 

A proprietary questionnaire was used for the study which had undergone a validation process (evaluation of the degree of reproducibility). The concordance of the two measurements was assessed by Cohen’s Kappa coefficient value which amounted to 0.82. Based on the adopted interpretation according to Landis and Koch, the result was interpreted as having “near-perfect” compliance [42].

The questionnaire consisted of 33 questions covering eating behaviour, non-food behaviour/physical activity, nutritional knowledge and a metric. The questionnaire included disjunctive-closed single-choice (the predominant part of the questionnaire), semi-open single-choice and open-ended short-answer questions. Two questions were constructed as closed-ended matrix questions in tabular form with the possibility of one answer per row [43,44]. The questionnaire in the English language is attached as a supplementary material to the article (Supplementary Material File S1).

### 2.3. Anthropometric Measurements and the Assessment of Children’s Nutritional Status

Anthropometric surveys were carried out at the premises of preschool facilities. The height (with an accuracy of 0.1 cm) and the weight (with an accuracy of 0.1 kg) were assessed among the children using the medical electronic personal scale “Seca 799” and the growth meter “Seca 220”. This was conducted in order to ensure the highest degree of reliability and validity with regard to the results based on the principles of anthropometric measurement as outlined in the device’s manual. Therefore, attention was made to ensure that the measuring tool was on a stable ground and its position was correctly regulated. In addition, during the evaluation of body height, the person being measured stood steadily, with their back and head erect, precisely under the callipers of the height gauge.

The children’s nutritional status was determined on the basis of their BMI value (BMI = body weight [kg]/body height [m^2^]). The obtained result was related to the growth standards dependent on a child’s gender and age developed by the WHO. Our analyses were based on the current WHO “*z*-scores” charts of BMI for age, which made it possible to establish the limits of underweight, normal body weight, overweight and obesity [45]. 

### 2.4. The Assessment of Parents’ Nutritional Knowledge

Parents’ knowledge of the nutrition of preschool children was assessed, among other things, on the basis of the following questions in the questionnaire of recommendations relating to the number of meals a child should eat; the source from which the largest percentage of energy in a child’s diet should come; a universal drink with which a child should quench their thirst; the number of portions of milk and dairy products; consumption of pulses; consumption of vegetables, fruit and meat and maximum salt intake. Attention was also drawn to the connection between nutrition and the correct development of the child and also the percentage of the fulfilment of daily energy requirements that meals consumed in the kindergarten should represent. Taking into account the respondents’ answers, a point scale was compiled. Criteria for assessing parents’ nutritional knowledge are shown in Table 1.

### 2.5. The Assessment of Parental Eating Behaviour

**Figure 2:**
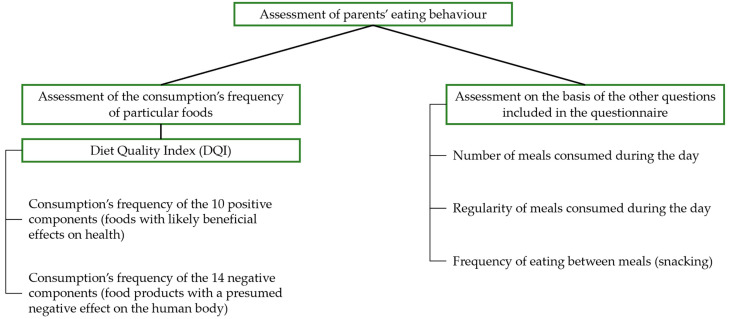
Assessment of parents’ eating behaviour.

Parents’ eating behaviour was assessed on the basis of the first part of the questionnaire (S1) in two ways according to Figure 2. On the basis of the question on the consumption frequency of individual products, the quality of the subjects’ diet was determined. For this purpose, the custom-transformed diet quality index (DQI) was used. This indicator identifies 24 food article groups, including 10 positive components (with a likely beneficial effect on health) and 14 negative ones which are presumed to have a negative effect on the human organism [46]. The responses obtained from the parents’ declared frequency of food product groups’ consumption were expressed as frequency indicators (multiplication rate/per day). The values obtained from the frequency of the products’ consumption were summed and, consequently, the DQI value was calculated for each parent using the following formula [46].

DQI (expressed in points) = (100/20) × sum of frequency of 10 food groups’ consumption [multiplication rate/per day] + (−100/28) × sum of frequency of 14 food groups’ consumption [multiplication rate/per day]

The final DQI score was related to the appropriate score ranges, allowing us to classify the quality of the parent’s diet into the categories of bad (−100 to −26 points), moderate (−25 to 25 points) and good (26 to 100 points). The remaining questions belonging to this section of the questionnaire were reviewed separately in the statistical analyses.

### 2.6. The Assessment of Parents’ Nutritional Status

The nutritional status was assessed on the basis of parents’ declared weight and body height in the proprietary questionnaire. Based on the obtained data, a BMI value was calculated for each subject. The classification of parents’ nutritional status was made taking into account the WHO guidelines available in the latest version of the ICD-11 (<18.50 kg/m^2^—underweight; 18.50–24.99 kg/m^2^—normal body weight; 25.00–29.99 kg/m^2^—overweight; 30.00–34.99 kg/m^2^—first degree obesity; 35.00–39.99 kg/m^2^—second degree obesity; ≥40.00 kg/m^2^—third degree obesity) [22].

### 2.7. The Statistical Methods of Processing the Results

Programs such as Microsoft Excel 2019 and Statistica 13.3 (StatSoft^®^, Tulsa, OK, USA) were used to statistically process the results. The Shapiro–Wilka W test was handled to check the normality of the distribution of variables. The distribution of variables deviated from normal, so non-parametric tests (Mann–Whitney U and Kruskal–Wallis) were resorted to in order to analyse the data. The Chi^2^ test was carried out to analyse the structure of the replies given by respondents in the author’s questionnaire depending on the selected factors. Tables of abundance, box plots (box-and-whisker diagrams 2W) and bar charts also served to statistically process the results. In each case, a *p*-value of ≤0.05 was taken as the level of significance (α = 0.05). The Spearman correlation coefficient was used to assess the relationship between the selected variables.

## 3. Results

### 3.1. The Characteristics of the Parents’ and Children’s Groups

The children’s carers were divided into two groups—girls’ and boys’ parents. Table 2 presents the characteristics of the parents, taking into account their age, weight and height as well as BMI value.

The children’s characteristics regarding their gender were presented in Table 3. Similarly, as with the parents, age, weight and height as well as BMI value were taken into account.

**Figure 3:**
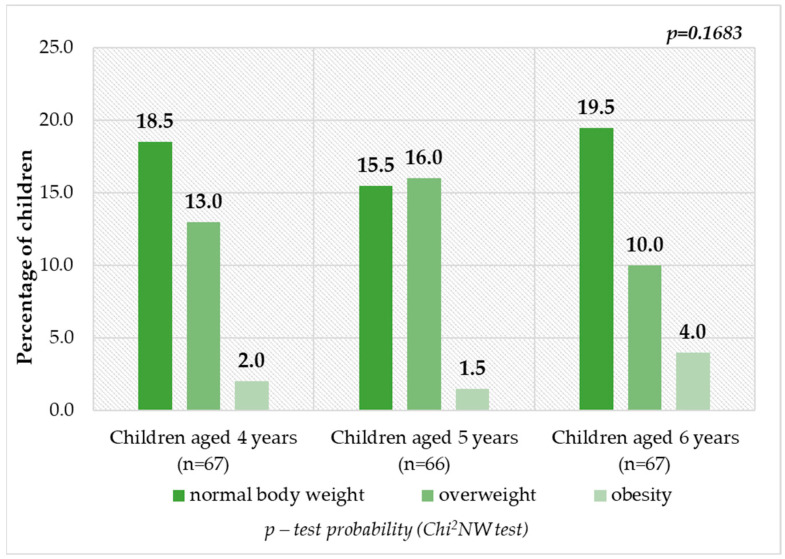
Children’s nutritional status by age group.

**Figure 4:**
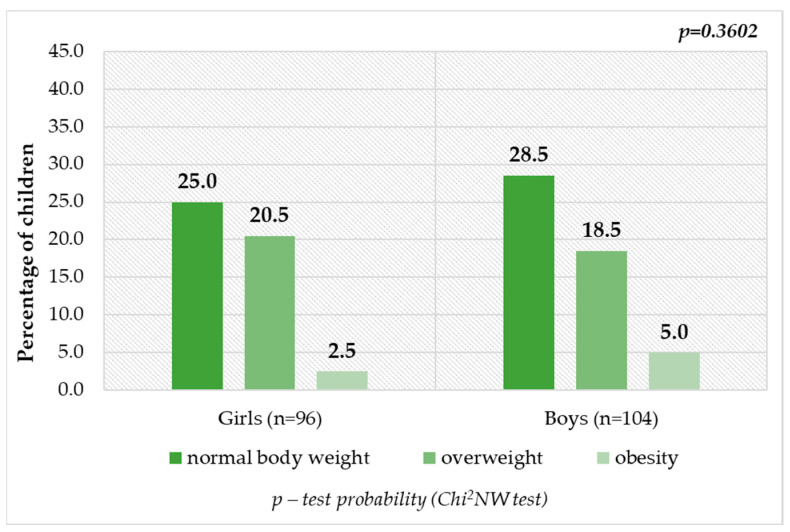
Children’s nutritional status by gender.

Figure 3 and Figure 4 show the children’s nutritional status according to their age and gender. Children at the age of 4, 5 and 6 years, whose BMI values were compatible with the norms, accounted for more than 50% of respondents in the indicated age groups, including 25.0% (*n* = 50) of girls and 28.5% (*n* = 57) of boys. The derogations from the correct values only included excessive body weight and thus underweight was not recognized among surveyed group of children. As many as 46.5% (*n* = 93) of preschool children were diagnosed as overweight or obese. This means that every second person was affected by the problem of excessive body weight. Overweight was detected in 26 children aged 4 years (13.0%), 32 children aged 5 years (16.0%) and 20 children aged 6 years (10.0%). On the other hand, obesity was diagnosed less frequently in four (2.0%), three (1.5%) and eight (4.0%) children (Figure 3), respectively. Excessive body weight was distinguished more often among boys than among girls (Figure 4). Nevertheless, differences between age groups and gender were not statistically significant (*p* = 0.1683 and *p* = 0.3602) (Figure 3 and Figure 4).

### 3.2. The Relationship between Parents’ Nutritional Knowledge and the Nutritional Status of Themselves and Their Children

**Figure 5:**
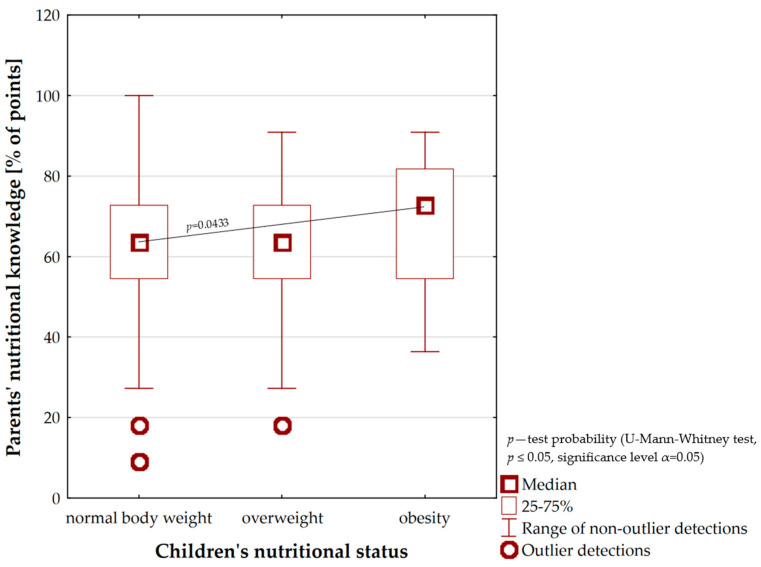
The relationship between parents’ nutritional knowledge and children’s nutritional status.

Figure 5 portrays the relationship between parents’ nutritional knowledge and children’s nutritional status. It was observed that as the level of parental knowledge increased, children were more likely to be overweight. The nutritional knowledge of parents, whose children were diagnosed with obesity, was at a good level (Me = 72.7% points). Overweight and normal body weight in minors were perceived when caregivers had a sufficient level of knowledge (Me = 63.6% points). Furthermore, it was noticed that minors’ parents with overweight or proper nutritional status showed a particularly insufficient level of knowledge about their children’s nutrition (*n* = 5). The percentage of points scored by them evolved between the levels of 9.0–18.0%. It was evidenced that the nutritional knowledge of girls’ and boys’ caregivers with obesity was significantly higher compared with children’s parents with a normal body weight (*p* = 0.0433). Additionally, on the basis of Spearman’s correlation coefficient, it was showed that a child’s BMI increased significantly with increasing parental nutritional knowledge (r = 0.23, *p* = 0.0011)

**Figure 6:**
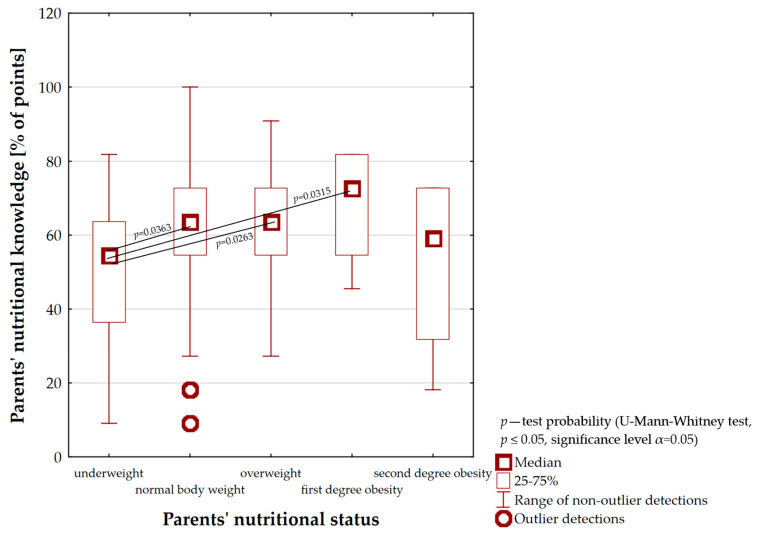
The relationship between parents’ nutritional knowledge and parents’ nutritional status.

In addition, an analysis of the relationship between parents’ nutritional knowledge and their nutritional status was carried out (Figure 6). It was observed that parents with a diagnosis of first degree obesity had the highest level of knowledge (Me = 72.7% of points) compared with other groups. Therefore, their knowledge of the principles of proper nutrition for children was defined as good. However, it is worth noting that only among parents with a normal body weight was the percentage of 100% for correct answers obtained. In the indicated group, there were also cases of caregivers whose knowledge was at an insufficient level. The nutritional knowledge of parents with diagnosed eating disorders (underweight, overweight and second degree obesity) was at the same sufficient level (respectively, Me = 54.5% of points, Me = 63.6% of points and Me = 59.1% of points). Moreover, the level of underweight parents’ nutritional knowledge was statistically significantly lower compared with normal body weight and overweight caregivers as well as those with first degree obesity (*p* = 0.0363, *p* = 0.0263 and *p* = 0.0315). Thus, it can be concluded that the same results were obtained for the relationship between parents’ nutritional knowledge and their children’s nutritional status—as the level of nutritional knowledge increased, BMI increased (to the first degree of obesity).

### 3.3. The Relationship between Parents’ Eating Behaviour and Their Children’s Nutritional Status

**Figure 7:**
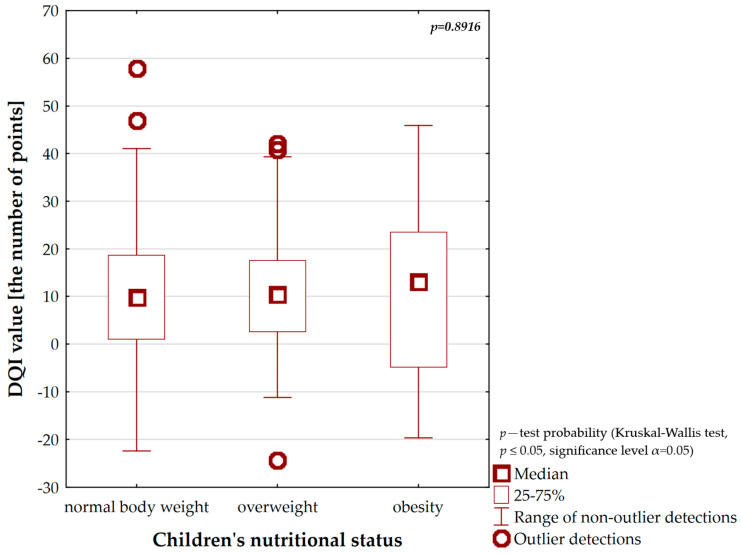
The relationship between DQI values and children’s nutritional status.

The relationship between the quality of a parent’s diet and a child’s nutritional status was included in Figure 7 and Table 4. The mid-point values of the DQI attested to the moderate quality of the parents’ diet (9.9–13.2 points). However, slightly higher DQI values were registered among caregivers whose children struggled with overweight (Me = 10.5 points) and obesity (Me = 13.2 points). However, it was perceived that in the group of children whose nutritional status was proper the parents followed the best (good) quality diet. By the same token, the DQI values oscillated between 47.0 and 57.9 points (Figure 7) among them. Among the surveyed parents with a good quality diet, cases of excessive body weight in their children were usually less frequently diagnosed versus caregivers with moderate diet quality. Overweight was determined, respectively, in 5.5% (*n* = 11) and 33.5% (*n* = 67) of children and obesity, respectively, in 1.0% (*n* = 2) and 6.5% (*n* = 13) of children (Table 4). In spite of this, these relationships were not statistically significant (Figure 7 and Table 4).

Supposedly, it is the number and regularity of eaten meals as well as the frequency with which parents eat between meals that may be closely related to their children’s eating behaviour [14,47,48]. 

This is also suggested by the results of our own study. It was noted that in the groups of parents that consumed the correct number of meals—consistent with the recommendations (from 4 to 5 per day)—the majority of children had a proper nutritional status (*n* = 69; 34.5%). On the other hand, overweight among children was mainly recorded when their caregivers declared eating three meals per day (*n* = 29; 14.5%). Obesity among children was repeatedly diagnosed when their parents claimed to eat between meals once per day (*n* = 5; 2.5%). Relatively often, the indicated disease was diagnosed among girls and boys whose caregivers consumed food between meals several times per day (*n* = 4; 2.0%) and several times per week (*n* = 3; 1.0%). In contrast, obesity was not present among minors whose caregivers acknowledged that they never snack. Nevertheless, overweight in children was observed when their parents declared snacking, respectively, several times per week (*n* = 30; 15.0%), several times per day (*n* = 20; 10.0%) and once per day (*n* = 16; 8.0%). Accordingly, as the parents’ frequency of eating between meals decreased, the number of overweight and obese children tended to decrease. However, the relationships described above were not statistically significant (*p* = 0.1007 and *p* = 0.505) (Table 5).

In Table 6, the relationship between parents’ consumption of meals at fixed times of the day and the child’s nutritional status was submitted. Excessive body weight was more common among children whose caregivers did not eat regularly or ate only some meals regularly. Overweight was diagnosed in 16.0% (*n* = 32) of children whose caregivers did not consume meals systematically and in 19.0% (*n* = 38) whose caregivers only consumed some meals regularly. Likewise, obesity was reported, respectively, in 3.0% (*n* = 6) and in 4.0% (*n* = 8) of children. The highest interest rate of children with a valid nutritional status (*n* = 55; 27.5%) was found in the group of parents who declared consuming only some meals regularly. The relationship that was described was statistically significant (*p* = 0.0049).

### 3.4. The Relationship between the Parent’s Nutritional Status and the Child’s Nutritional Status

The relationship between the parent’s nutritional status and the child’s nutritional status based on the obtained BMI values is shown in Table 7. On the basis of Spearman’s correlation coefficient, it was shown that as the parents’ BMI values increased, their children’s BMI values increased in a way that was statistically significantly (r = 0.25; *p* = 0.0003). Therefore, it was found that the highest percentage of children (36.0%) with a correct nutritional status was found in the group of parents whose BMI was consistent with the norms. The excessive body weight that was recognized among parents correspondingly predisposed the development of these disorders in minors. Parents’ children whose BMI was indicative of overweight were burdened by excessive body weight at a percentage of 15.0% (overweight at 11.0% and obesity at 4.0%). Caregivers’ children with first and second degree obesity struggled with overweight in 3.0% and obesity in 1.5%. To the lowest extent, the problem of excessive body weight affected the children of underweight parents. Only among 1% of girls and boys in the above-mentioned group were diagnosed as overweight, whereas there was no child in the studied group that was diagnosed as obese. The relationships between the parent’s and the child’s nutritional status was statistically significant (*p* = 0.0070).

### 3.5. The Relationships between Sociodemographic Factors and the Child’s and the Parent’s Nutritional Status 

Table 8 shows the child’s and the parent’s nutritional status according to the education level, place of residence and professional status of the parent. It was noted that the parents’ nutritional status was significantly influenced by place of residence (*p* = 0.0192). Therefore, excessive body weight was diagnosed more frequently among groups of parents living in rural areas and cities with up to 100,000 inhabitants.

The child’s nutritional status was statistically significantly influenced by the parents’ level of education (*p* = 0.0379). Thus, in the community of caregivers who indicated a higher education level, a predominant percentage of children with normal body weight was observed (*n* = 58; 29.0%). Parents’ children with a secondary level of education were the second most numerous group in which no deviation from the standards in terms of BMI values was observed (*n* = 33; 16.5%). In contrast, among parents’ children with a primary level of education, overweight or obesity were more frequently diagnosed than body weight in line with the norms. 

### 3.6. The Relationships between Sociodemographic Factors and Parental Knowledge and Eating Behaviour 

The knowledge of more than half of the parents (53.0%) regarding their children’s nutrition was ranked at a sufficient level, which suggests a need for further nutrition education. A statistically significant association was observed between education level and nutrition knowledge (*p* = 0.0206). The higher the level of education the better the parents’ knowledge. In addition, the group with a higher educational level reported parents who showed very good knowledge of child nutrition. Statistically significant associations were not observed between parents’ nutritional knowledge and their place of residence as well as professional status (Table 9).

Table 9 also shows the associations between parental diet quality and specified sociodemographic factors. Significant relationships were only proved between the DQI and educational level (*p* = 0.0296). Among parents declaring a higher level of education, the highest percentage of those with good diet quality (11.5%) was registered, while only 0.5% of such people were recorded in the primary and vocational education groups.

## 4. Discussion

The aim of the undertaken study was the assessment of the relationship between parental knowledge and dietary behaviour and the prevalence of overweight and obesity in preschool children. Furthermore, the purpose of the study was to appraise the prevalence of excessive body weight in the indicative age group. The hypothesis adopted at the outset was the statement that “parents’ inadequate knowledge and their inappropriate eating behaviour are significantly associated with the prevalence of excessive body weight among preschool children” was partially confirmed alongside other research’s assumptions. Compliance was obtained in the context of the association between inappropriate eating behaviour and an increased risk of developing overweight and obesity among minors. In contrast, an innovative association was observed within the influence of increased parental nutritional knowledge on the prevalence of excessive body weight in preschool children.

Excessive body weight is an increasingly serious problem that is already affecting the youngest part of the population. Both overweight and obesity, as disease units, constitute a particular danger to preschool children [49,50]. The crucial role during this period is attributed to parents as they are the ones most responsible for devising their children’s eating habits and preferences. An equally important aspect is that the diet and health behaviour of caregivers are most often adopted by children and perpetuated into the later years of their lives [51,52].

As was mentioned above, the relationship between parental knowledge and dietary behaviour and the prevalence of overweight and obesity among children are the subject of research among many authors. In studies, researchers also draw attention to the continuing rise in the incidence of overweight and obesity among the youngest part of the population [25,26,27,28,29,30,32,33,53]. 

On the basis of our own study, the statistically significant relationship between parents’ nutritional knowledge and their children’s nutritional status was shown. The obtained results are paradoxical because as the level of nutritional knowledge of parents escalated the number of overweight and obese children increased (Figure 5). Therefore, it can be assumed that parents’ nutritional knowledge is not put into practice. This may be related to the lack of carers’ time to prepare valuable meals for their children, which results in a preference for ready-made (instant) meals including highly processed products or fast food. This type of food is characterised by low nutritional value and promotes the development of excessive body weight. The above-mentioned aspects were highlighted in the study developed by Kim et al. (2019) [53]. The authors additionally pointed out that other family members—including grandparents—often intervene in children’s nutrition. Generational differences repeatedly led to inconsistencies in the formation of appropriate eating behaviour among the youngest and to different perceptions of the “unhealthy” food concept. Our own findings, insinuating that higher levels of parental nutritional knowledge predispose the development of overweight and obesity in children, are somewhat in contrast to studies carried out by other authors. Indeed, a study conducted by Jarosz (2016) found that an increase in the level of caregivers’ knowledge was associated with a decreasing number of children affected by eating disorders [25]. Similar results were obtained in the study carried out by Vereecken and Maes (2010) who showed that mothers’ nutritional knowledge positively influenced children’s eating behaviour, including the appropriate quality of their diet [26]. Analyses implemented through 302 mothers from Ankara by Yabancı et al. (2014) also demonstrated that higher nutritional knowledge was more often associated with normal nutritional status among children [27]. This is equivalent to the fact that children, whose mothers showed better knowledge of nutrition, were characterised by a normal body weight. In contrast, other analyses presented a different point of view with regard to the positions above. The investigators indicated that there was no relationship between parents’ nutritional knowledge and the consumption of healthy foods and thus the maintenance of a proper state of health for children. They also stressed that an appropriate level of parental nutritional knowledge was not the sole factor in the preservation of children’s body weight. For that reason, the results of our own study constitute a new perspective on parental influence on the occurrence of excessive body weight among preschool children [28,29,30].

Researchers repeatedly emphasise the impact of parents’ eating behaviour on children’s nutritional status in their studies. The significant role of carers, especially mothers, is that they are responsible for preparing meals at home. In practice, parents decide on the quality of the food their child eats [41,53]. It is also highlighted that inappropriate parental dietary behaviour, including habits and routines, leads to weight gain in children due to abnormalities perpetuated over the years [31]. Thus, the youngest usually adopt the erroneous dietary style of their caregivers, who are their main role models. Based on the analyses by Demir and Bektas (2017), it was shown that parents’ behaviour and eating style were the cause of obesity among 19% of the surveyed children [32]. 

The authors emphasised that one of the abnormal behaviours that promotes the development of excessive body weight is the lack of regular meal consumption [33]. Therefore, it can be supposed that there exists a high probability of irregular meal consumption by all family members. Our own analyses exposed a statistically significant relationship between the regularity of meals eaten by caregivers and the prevalence of overweight and obesity among their children (*p* = 0.0049) (Table 6). Parents’ children who declared a lack of consumption of meals at regular times during the day were more likely to be laden with excessive body weight. The consumption of all meals at fixed times of the day was indicated by only 19.0% of respondents. The comparable distribution of data was noted in the study by Newerli-Guz and Kulwikowska (2014) in which systematic meal consumption was registered in only 15.0% of parents. The issue of regular meal consumption can be considered as one of the most significant forms of nonconformity within carers’ eating behaviour, predisposing the development of excessive body weight among children [54].

Mushonga et al. (2017) also identified that children skipping the consumption of breakfast to be an inappropriate eating behaviour affecting the evolution of overweight and obesity among minors [55]. Furthermore, the meta-analyses by Yee et al. (2017) showed a strong correlation between parents’ and their children’s eating behaviour in terms of the child’s adoption of the desire to eat healthy foods as well as those with post-tenuous negative effects on the body [34]. The cited matter can be considered with reference to the increased risk of developing excessive body weight in children whose diets would be rich in products that contribute to the deterioration of their nutritional status.

In addition, researchers show that minors largely take on the dietary patterns of their parents, which can directly influence the development of abnormalities among girls and boys including excessive body weight [35]. Mistry and Puthussery (2015), in a study conducted in South Asian countries, stated that one of the main risk factors for the evolution of overweight and obesity among children was the family burden of these diseases [36]. In the analysis undertaken by Kołpa et al. (2017), it was visible that caregivers of overweight children were also laden with these disorders at almost 37% [37]. Stąpor et al. (2016) also demonstrated that excessive body weight diagnosed in children’s carers was closely related to the prevalence of these disorders among minors [38]. Additionally, the researchers emphasised that the consumption of meals at home, in the sphere of the family promoted the formation of appropriate eating behaviour from an early age [56]. Our own examination also revealed that the nutritional status of children is dependent on the nutritional status of their parents (*p* = 0.0070). Parents’ children, whose BMI indicated overweight or obesity, were burdened with excessive body weight at a percentage of 19.5% (Table 7). What is more, our own study showed that a child’s nutritional status is statistically significantly influenced by the parents’ level of education (*p* = 0.0379) (Table 8). The identical relationship was reported in a study conducted by Parikka et al. (2015). The researchers proved that a lower level of parental education was associated with a higher prevalence of excessive body weight among the children’s surveyed group [57].

The associations between sociodemographic factors and parents’ level of nutritional knowledge are also the subject of numerous studies. In the analysis of Jarosz (2016), a significant relation was noted between the respondents’ declared education and the assessed level of nutritional knowledge [25]. The indicative above-mentioned statistically significant relationship (*p* = 0.028) was also highlighted in a study conducted by Mushonga et al. (2017) in the area of Zimbabwe among 241 preschool children’s parents [55]. Furthermore, a study which was carried out by Bissinger (2020) among 126 women showed that as the educational level of the surveyed units increased, their nutritional knowledge increased [58]. Nevertheless, the afore-cited analysis did not reveal any influence from demographic factors on the respondents’ state of knowledge. The same results were registered in the case of our own study, which allows us to conclude that education to the greatest degree determines the respondents’ level of knowledge about the children’s nutrition.

The outcomes of our own examination and the results of the other authors’ analyses compared with them confirm that excessive body weight is a highly prevalent problem among preschool children. Furthermore, this point is intently linked to parents’ knowledge and eating behaviour. Nonetheless, our own analyses lead to a partial confirmation of the research hypothesis that was established at the outset and states that parents’ insufficient level of knowledge and their inappropriate eating behaviour have a significant impact on the prevalence of overweight and obesity among preschool children.

### Strengths and Limitations of the Study

The results obtained in this study can contribute to the development of actions that are taken by professionals in the area of public health, designed to increase nutritional knowledge and change the nutritional behaviour of families. Furthermore, according to our knowledge, this is the first study that more broadly defines the association of nutritional knowledge, dietary behaviour, diet quality and parents’ nutritional status with the risk of nutritional abnormalities among preschool children. The authors’ index of nutritional knowledge was created for this study, adapted for a self-administered questionnaire and provides the groundwork for describing the original methodology. Furthermore, the parents’ eating behaviour was assessed in two ways through separately considering the quality of the respondents’ diet (using the DQI) and their eating behaviour (e.g., the regularity of meals eaten during the day). In addition, the influence of sociodemographic factors on the above-mentioned variables was also taken into account. It should be emphasised that this is a pilot study carried out in one voivodship in Poland. Therefore, it is not possible to take the results of this survey as general fallout of the entire population of Poland as well as other countries inter alia because of cultural and economic differences. In addition, due to the pilot character of the study, it was examined and checked in a general way whether the relationships identified in the title of the article exist. This undoubtedly entails additional limitations, inter alia not taking into account the dietary practices of parents for their children (including the time spent on meal preparation) and the eating habits of minors. The state of knowledge in the field of this issue could be further developed by research focused on the extent to which children are fed by family members other than their parents including those living in the household with them (for instance, grandparents). In addition, our own research concentrated mainly on the matter related to the consumption frequency of specific food groups by parents, while it was not connected with the quantity of items consumed by caregivers. This is also a crucial aspect in the development of excessive body weight and can be adopted by children. Furthermore, the study could be extended to include additional socioeconomic factors that may influence the prevalence of excessive body weight among preschool children such as, for instance, parents’ income (material situation) and marital status or the nature of the carers’ work. However, the lack of reference for the indicated elements and relationships is justified by the fact that the developed questionnaire was intended to be dedicated as much as possible to the selected group of respondents (parents of preschool children). Therefore, the questionnaire was prepared in such a way that the time needed to complete it oscillated by approximately 10 min. It was taken into account that a longer time spent on filling in the form could lead to the fatigue of the respondent and thus a lower quality of the obtained results. This is confirmed by researchers cited by Sharma (2022) [59]. The obtained results of our own study can be considered as a basis for further investigation into the problem, especially taking into account the paradoxical relationship that better nutritional knowledge among parents did not result in a reduction in the prevalence of excessive body weight among children.

Notwithstanding, the study provides valuable information about the association of parental knowledge and dietary behaviour with the prevalence of overweight and obesity among preschool children. Further studies covering other regions of Poland as well as other countries are needed to obtain more reliable results in this field. The present article pays attention to the increasing problem of excessive body weight in recent years. Disease entities such as overweight and obesity are particularly dangerous for children who, due to their own state of psychophysical development, are unable to diagnose and analyse harmful factors in terms of nutrition. Accordingly, reducing the risk of the above-mentioned diseases is a challenge not only for parents but also for dieticians, nutrition specialists and nutrition educators. The issue of excessive body weight can certainly not be underestimated because increasing levels of consumption thoroughly promote nutritional disorders whose consequences can be serious for health and even life.

## 5. Conclusions

On the basis of the conducted study, it was concluded that parental knowledge and dietary behaviour are related to the phenomenon of excessive body weight among children. The recorded increase in the incidence of overweight and obesity among minors whose parents exhibit a higher level of nutritional knowledge leads to the assumption that there are some discrepancies between the practical and theoretical approaches to this issue. Caregivers should pay particular attention to the regularity of the meals consumed by them in as much as the indicated eating behaviour is the most likely to be adopted by children. Indeed, it has been confirmed that abnormalities in this area imply the development of excessive body weight among preschool girls and boys. There is a further necessity to educate parents about nutrition as they play a significant role in moulding the nutritional status of minors. The elimination of dietary malformations from the earliest years of life can contribute to maintaining health in both the present and the future.

The conducted research encourages increased interest in the issue of overweight and obesity among preschool children, particularly in the field of dietetics and nutrition science. It becomes important to extend the analysis to other additional factors that also have a crucial impact on the increased risk of developing excessive body weight. The current situation, associated with an intensive growth in the incidence of overweight and obesity among the youngest members of society especially, poses new challenges in the presented research fields, making it necessary to develop innovative methods and approaches for prevention and therapy.

## Figures and Tables

**Table 1:** Assessment of parents’ nutritional knowledge according to the author’s nutrition knowledge index.

Method of Scoring Answers Given by Parents			
Correct answer		1 point	
Incorrect answer		0 points	
**Assessment of Parents’** **Nutritional Knowledge**	**Numbers of** **Obtained Points**		**Percentage of** **Obtained Points**
Insufficient	0.0–4.0		<45%
Sufficient	5.0–7.0		<45–64%
Good	8.0–9.0		<64–90%
Very good	10.0–11.0		<90–100%

**Table 2:** The characteristics of the study group (girls’ and boys’ parents).

Characteristics		Total *n* = 200	Parents’ Group		*p*
			Girls’ Parents *n* = 96	Boys’ Parents *n* = 104	
Age [years]	$\bar{x}$ ± SD	35.0 ± 5.7	35.0 ± 5.8	35.0 ± 5.5	0.1526
	Me [Q1;Q3]	35.0 [32.0;39.0]	36.0 [32.0;39.0]	35.0 [31.0;38.0]	
	Min–Max	23.0–56.0	23.0–56.0	24.0–55.0	
Body weight [kg]	$\bar{x}$ ± SD	66.6 ± 12.5	66.3 ± 12.4	66.9 ± 12.6	0.8059
	Me [Q1;Q3]	65.0 [58.0;71.5]	65.0 [58.5;70.5]	65.0 [58.0;72.0]	
	Min–Max	46.0–125.0	46.0–125.0	47.0–110.0	
Body height [cm]	$\bar{x}$ ± SD	166.0 ± 6.7	165.1 ± 6.9	166.9 ± 6.4	0.0207
	Me [Q1;Q3]	165.0 [160.5;170.0]	164.5 [160.0;168.5]	166.0 [163.0;170.0]	
	Min–Max	149.0–185.0	152.0–185.0	149.0–185.0	
Body Mass Index (BMI) [kg/m^2^]	$\bar{x}$ ± SD	24.1 ± 3.9	24.3 ± 3.8	24.0 ± 3.9	0.4587
	Me [Q1;Q3]	23.4 [21.8;25.8]	24.0 [22.4;26.0]	23.3 [21.7;25.5]	
	Min–Max	16.4–36.5	16.6–36.5	16.4–34.7	

*n*—sample size; $\bar{x}$—average; SD—standard deviation; Me—median; Q1—lower quartile; Q3—upper quartile; Min—minimum value; Max—maximum value; *p*—test probability (Mann–Whitney U test, *p* ≤ 0.05, significance level α = 0.05).

**Table 3:** The characteristics of the children’s study group.

Characteristics		Total *n* = 200	Gender of the Child		*p*
			Girls *n* = 96	Boys *n* = 104	
Age [years]	$\bar{x}$ ± SD	5.0 ± 0.8	5.0 ± 0.9	5.0 ± 0.8	0.5154
	Me [Q1;Q3]	5.0 [4.0;6.0]	5.0 [4.0;6.0]	5.0 [4.0;6.0]	
	Min–Max	4.0–6.0	4.0–6.0	4.0–6.0	
Body weight [kg]	$\bar{x}$ ± SD	20.2 ± 4.1	20.3 ± 4.1	20.0 ± 4.2	0.5233
	Me [Q1;Q3]	19.6 [17.1;22.4]	19.8 [17.1;22.9]	19.5 [17.1;22.1]	
	Min–Max	12.4–34.2	14.6–34.2	12.4–33.4	
Body height [cm]	$\bar{x}$ ± SD	108.7 ± 8.7	108.9 ± 8.7	108.5 ± 8.7	0.7330
	Me [Q1;Q3]	108.0 [102.5;115.2]	108.7 [102.2;115.4]	107.9 [102.8;114.8]	
	Min–Max	88.1–132.3	91.9–132.3	88.1–131.5	
Body Mass Index (BMI) [kg/m^2^]	$\bar{x}$ ± SD	17.0 ± 2.0	17.0 ± 2.1	16.9 ± 1.8	0.5639
	Me [Q1;Q3]	16.6 [15.7;17.6]	16.9 [15.8;17.7]	16.5 [15.7;17.6]	
	Min–Max	13.0–27.3	13.0–27.3	13.8–22.9	

*n*—sample size; $\bar{x}$—average; SD—standard deviation; Me—median; Q1—lower quartile; Q3—upper quartile; Min—minimum value; Max—maximum value; *p*—test probability (Mann–Whitney U test, *p* ≤ 0.05, significance level α = 0.05).

**Table 4:** The relationship between the quality of the parent’s diet and the child’s nutritional status.

Quality of the Parent’s Diet	Parameter	Child’s Nutritional Status			Total	*p*
		Normal Body Weight	Overweight	Obesity		
Moderate	*n*	90	67	13	170	0.9282
	% of column	84.1	85.9	86.7	-	
	% of row	52.9	39.4	7.7	-	
	% of total	45.0	33.5	6.5	85.0	
Good	*n*	17	11	2	30	
	% of column	15.9	14.1	13.3	-	
	% of row	56.7	36.7	6.7	-	
	% of total	8.5	5.5	1.0	15.0	
Total		107	78	15	200	
%		53.5	39.0	7.5	100.0	

*n*—sample size; *p*—test probability (Chi^2^NW test, *p* ≤ 0.05, significance level α = 0.05).

**Table 5:** Associations between parental eating behaviour and child’s nutritional status.

Parents’ Eating Behaviour	Parameter	Child’s Nutritional Status			Total	*p*
		Normal Body Weight	Overweight	Obesity		
Number of meals consumed during the day						
1 meal	*n*	0	1	0	1	0.1007
	%	0.0	0.5	0.0	0.5	
2 meals	*n*	8	3	1	12	
	%	4.0	1.5	0.5	6.0	
3 meals	*n*	29	29	4	62	
	%	14.5	14.5	2.0	31.0	
4 meals	*n*	52	27	3	82	
	%	26.0	13.5	1.5	41.0	
5 meals	*n*	17	15	7	39	
	%	8.5	7.5	3.5	19.5	
Above 5 meals	*n*	1	3	0	4	
	%	0.5	1.5	0.0	2.0	
Frequency of eating between meals (snacking)						
Several times per day	*n*	13	16	4	22	0.5053
	%	6.5	8.0	2.0	16.5	
Once per day	*n*	34	20	5	59	
	%	17.0	10.0	2.5	29.5	
Several times per week	*n*	34	30	3	67	
	%	17.0	15.0	1.5	33.5	
Once per week	*n*	11	5	2	18	
	%	5.5	2.5	1.0	9.0	
1–3 times per month	*n*	8	5	1	14	
	%	4.0	2.5	0.5	7.0	
Never	*n*	7	2	0	9	
	%	3.5	1.0	0.0	4.5	

*n*—sample size; *p*—test probability (Chi^2^NW test, *p* ≤ 0.05, significance level α = 0.05).

**Table 6:** The relationship between the parent’s consumption of meals at regular times of the day and the child’s nutritional status.

Eating at Regular Times during the Day	Parameter	Child’s Nutritional Status			Total	*p*
		Normal Body Weight	Overweight	Obesity		
No	*n*	23	32	6	61	0.0049
	% of total	11.5	16.0	3.0	30.5	
Yes, but only certain	*n*	55	38	8	101	
	% of total	27.5	19.0	4.0	50.5	
Yes	*n*	29	8	1	38	
	% of total	14.5	4.0	0.5	19.0	
Total		107	78	15	200	
%		53.5	39.0	7.5	100.0	

*n*—sample size; *p*—test probability (Chi^2^NW test, *p* ≤ 0.05, significance level α = 0.05).

**Table 7:** The relationship between the parent’s and the child’s nutritional status.

Nutritional Status of the Child	Parameter	Nutritional Status of the Parent					Total	*p*
		Underweight	Normal Body Weight	Overweight	First Degree Obesity	Second Degree Obesity		
Normal body weight	*n*	10	72	18	5	2	107	0.0070
	% of total	5.0	36.0	9.0	2.5	1.0	53.5	
Overweight	*n*	2	48	22	4	2	78	
	% of total	1.0	24.0	11.0	2.0	1.0	39.0	
Obesity	*n*	0	4	8	3	0	15	
	% of total	0.0	2.0	4.0	1.5	0.0	7.5	
Total		12	124	48	12	4	200	
%		6.0	62.0	24.0	6.0	2.0	100.0	

*n*—sample size; *p*–test probability (Chi^2^NW test, *p* ≤ 0.05, significance level α = 0.05).

**Table 8:** Child’s and parent’s nutritional status in accordance with education, place of residence and parent’s professional status.

The Socio- Demographic Factor	Parameter	Child’s Nutritional Status			Total	*p*	Parent’s Nutritional Status					Total	*p*
		Normal Body Weight	Overweight	Obesity			Underweight	Normal Body Weight	Overweight	First Degree Obesity	Second Degree Obesity		
Education													
Primary	*n*	2	3	2	7	0.0379	0	5	2	0	0	7	0.6595
	%	1.0	1.5	1.0	3.5		0.0	2.5	1.0	0.0	0.0	3.5	
Secondary	*n*	33	27	1	61		5	39	11	4	2	61	
	%	16.5	13.5	0.5	30.5		2.5	19.5	5.5	2.0	1.0	30.5	
Professional	*n*	14	6	5	25		2	11	10	2	0	25	
	%	7.0	3.0	2.5	12.5		1.0	5.5	5.0	1.0	0.0	12.5	
Higher	*n*	58	42	7	107		5	69	25	6	2	107	
	%	29.0	21.0	3.5	53.5		2.5	34.5	12.5	3.0	1.0	53.5	
Place of residence													
Village	*n*	13	8	5	26	0.1015	0	19	2	5	0	26	0.0192
	%	6.5	4.0	2.5	13.0		0.0	9.5	1.0	2.5	0.0	13.0	
City of up to 100,000 inhabitants	*n*	81	66	9	156		10	94	41	7	4	156	
	%	40.5	33.0	4.5	78.0		5.0	47.0	20.5	3.5	2.0	78.0	
City with more than 100,000 inhabitants	*n*	13	4	1	18		11	11	5	0	0	18	
	%	6.5	2.0	0.5	9.0		5.5	5.5	2.5	0.0	0.0	9.0	
Professional status													
Student	*n*	0	1	0	1	0.7132	0	1	0	0	0	1	0.6450
	%	0.0	0.5	0.0	0.5		0.0	0.5	0.0	0.0	0.0	0.5	
Working	*n*	92	66	13	171		9	108	40	10	4	171	
	%	46.0	33.0	6.5	85.5		4.5	54.0	20.0	5.0	2.0	85.5	
Unemployed	*n*	12	6	2	20		2	11	6	1	0	20	
	%	6.0	3.0	1.0	10.0		1.0	5.5	3.0	0.5	0.0	10.0	
On a pension	*n*	1	1	0	2		1	0	0	1	0	2	
	%	0.5	0.5	0.0	1.0		0.5	0.0	0.0	0.5	0.0	1.0	
Other	*n*	2	4	0	6		0	4	2	0	0	6	
	%	1.0	2.0	0.0	3.0		0.0	2.0	1.0	0.0	0.0	3.0	

*n*—sample size; *p*—test probability (Chi^2^NW test, *p* ≤ 0.05, significance level α = 0.05).

**Table 9:** DQI and parents’ level of nutritional knowledge according to their education, place of residence and professional status.

The Socio- Demographic Factor	Parameter	Assessment of Parent’s Diet Quality Based on DQI		Total	*p*	Assessment of Parent’s Nutritional Knowledge				Total	*p*
		Moderate	Good			Insufficient	Sufficient	Good	Very Good		
Education											
Primary	*n*	6	1	7	0.0296	1	4	2	0	7	0.0206
	%	3.0	0.5	3.5		0.5	2.0	1.0	0.0	3.5	
Secondary	*n*	56	5	61		10	32	18	1	61	
	%	28.0	2.5	30.5		5.0	16.0	9.0	0.5	30.5	
Professional	*n*	24	1	25		4	16	5	0	25	
	%	12.0	0.5	12.5		2.0	8.0	2.5	0.0	12.5	
Higher	*n*	84	23	107		3	54	43	7	107	
	%	42.0	11.5	53.5		1.5	27.0	21.5	3.5	53.5	
Place of residence											
Village	*n*	21	5	26	0.3764	4	11	9	2	26	0.3683
	%	10.5	2.5	13.0		2.0	5.5	4.5	1.0	13.0	
City of up to 100,000 inhabitants	*n*	132	24	156		11	84	55	6	156	
	%	66.0	12.0	78.0		5.5	42.0	27.5	3.0	78.0	
City with more than 100,000 inhabitants	*n*	17	1	18		3	11	4	0	18	
	%	8.5	0.5	9.0		1.5	5.5	2.0	0.0	9.0	
Professional status											
Student	*n*	1	0	1	0.8256	0	1	0	0	1	0.1508
	%	0.5	0.0	0.5		0.0	0.5	0.0	0.0	0.5	
Working	*n*	144	27	171		11	89	63	8	171	
	%	72.0	13.5	85.5		5.5	44.5	31.5	4.0	85.5	
Unemployed	*n*	18	2	20		6	11	3	0	20	
	%	9.0	1.0	10.0		3.0	5.5	1.5	0.0	10.0	
On a pension	*n*	2	0	2		0	2	0	0	2	
	%	1.0	0.0	1.0		0.0	1.0	0.0	0.0	1.0	
Other	*n*	5	1	6		1	3	2	0	6	
	%	2.5	0.5	3.0		0.5	1.5	1.0	0.0	3.0	

*n*—sample size; *p*—test probability (Chi^2^NW test, *p* ≤ 0.05, significance level α = 0.05).

## Data Availability

Data are contained within the article and Supplementary Materials.

## Supplementary Materials

The following supporting information can be downloaded at: https://www.mdpi.com/article/10.3390/nu16010174/s1, File S1: Questionnaire to investigate the knowledge and eating behaviour of preschool aged children’s parents.

## References

[1] Ofori-Asenso R., Agyeman A.A., Laar A., Boateng D. (2016). Overweight and obesity epidemic in Ghana—A systematic review and meta-analysis. BMC Public Health.

[2] World Obesity Federation (2023). World Obesity Atlas 2023.

[3] World Health Organization (2022). World Obesity Day 2022—Accelerating Action to Stop Obesity.

[4] Garrido-Miguel M., Oliveira A., Cavero-Redondo I., Álvarez-Bueno C., Pozuelo-Carrascosa D.P., Soriano-Cano A., Martínez-Vizcaíno V. (2019). Prevalence of Overweight and Obesity among European Preschool Children: A Systematic Review and Meta-Regression by Food Group Consumption. Nutrients.

[5] World Obesity Day (2023). Changing Perspectives: Let’s Talk about Obesity. WOD. https://www.worldobesityday.org/.

[6] Bygdell M., Célind J., Lilja L., Martikainen J., Simonson L., Sjögren L., Ohlsson C., Kindblom J.M. (2021). Prevalence of overweight and obesity from 5 to 19 years of age in Gothenburg, Sweden. Acta Paediatr..

[7] Cunningham S.A., Kramer M.R., Venkat Narayan K.M. (2014). Incidence of Childhood Obesity in the United States. N. Engl. J. Med..

[8] Llewellyn A., Simmonds M., Owen C.G., Woolacott N. (2016). Childhood obesity as a predictor of morbidity in adulthood: A systematic review and meta-analysis. Obes. Rev..

[9] Sahoo K., Sahoo B., Choudhury A.K., Sofi N.Y., Kumar R., Bhadoria A.S. (2015). Childhood obesity: Causes and consequences. J. Fam. Med. Prim. Care.

[10] Klingelhöfer D., Braun M., Quarcoo D., Brüggmann D., Groneberg D.A. (2021). Epidemiological Influences and Requirements of Global Childhood Obesity Research. Obes. Facts.

[11] Androutsos O., Perperidi M., Georgiou C., Chouliaras G. (2021). Lifestyle Changes and Determinants of Children’s and Adolescents’ Body Weight Increase during the First COVID-19 Lockdown in Greece: The COV-EAT Study. Nutrients.

[12] Al Hourani H., Alkhatib B., Abdullah M. (2022). Impact of COVID-19 Lockdown on Body Weight, Eating Habits, and Physical Activity of Jordanian Children and Adolescents. Disaster Med. Public Health Prep..

[13] Weihrauch-Blüher S., Kromeyer-Hauschild K., Graf C., Widhalm K., Korsten-Reck U., Jödicke B., Markert J., Müller M.J., Moss A., Wabitsch M. (2018). Current Guidelines for Obesity Prevention in Childhood and Adolescence. Obes. Facts.

[14] Mahmood L., Flores-Barrantes P., Moreno L.A., Manios Y., Gonzalez-Gil E.M. (2021). The Influence of Parental Dietary Behaviors and Practices on Children’s Eating Habits. Nutrients.

[15] Hoffmann D.A., Marx J.M., Kiefner-Burmeister A., Musher-Eizenman D.R. (2016). Influence of maternal feeding goals and practices on children’s eating behaviors. Appetite.

[16] Pamungkas R.A., Chamroonsawasdi K. (2019). Home-Based Interventions to Treat and Prevent Childhood Obesity: A Systematic Review and Meta-Analysis. Behav. Sci..

[17] Balantekin K.N., Anzman-Frasca S., Francis L.A., Ventura A.K., Fisher J.O., Johnson S.L. (2020). Positive parenting approaches and their association with child eating and weight: A narrative review from infancy to adolescence. Pediatr. Obes..

[18] Scaglioni S., De Cosmi V., Ciappolino V., Parazzini F., Brambilla P., Agostoni C. (2018). Factors Influencing Children’s Eating Behaviours. Nutrients.

[19] Martins C., Norsett-Carr A. (2018). Obesity Knowledge among Final-Year Medical Students in Norway. Obes. Facts.

[20] Christensen S. (2020). Recognizing obesity as a disease. J. Am. Assoc. Nurse Pract..

[21] Hemmingsson E. (2018). Early Childhood Obesity Risk Factors: Socioeconomic Adversity, Family Dysfunction, Offspring Distress, and Junk Food Self-Medication. Curr. Obes. Rep..

[22] World Health Organization (2023). International Classification of Diseases 11th. ICD-11 for Mortality and Morbidity Statistics (ICD-11 MMS).

[23] La Sala L., Pontiroli A.E. (2020). Prevention of Diabetes and Cardiovascular Disease in Obesity. Int. J. Mol. Sci..

[24] Lee E.Y., Yoon K.H. (2018). Epidemic obesity in children and adolescents: Risk factors and prevention. Front. Med..

[25] Jarosz K. (2016). Evaluation of Selected Anthropometric Parameters of Preschool Children and the Level of Nutritional Knowledge of Their Parents. Master’s Thesis.

[26] Vereecken C., Maes L. (2010). Young children’s dietary habits and associations with the mothers’ nutritional knowledge and attitudes. Appetite.

[27] Yabancı N., Kısaç İ., Karakuş S.S. (2014). The effects of mother’s nutritional knowledge on attitudes and behaviors of children about nutrition. Procedia Soc. Behav. Sci..

[28] Almulla A.A., Alanazi A.S., Khasawneh M.A.S. (2023). The Relationship between Nutritional Intake and Mother’s Education Level with the Nutritional Status of Children with Special Needs. J. ReAttach Ther. Dev. Divers..

[29] El-Nmer F., Salama A.A., Elhawary D. (2014). Nutritional knowledge, attitude, and practice of parents and its impact on growth of their children. Menoufia Med. J..

[30] Romanos-Nanclares A., Zazpe I., Santiago S., Marín L., Rico-Campà A., Martín-Calvo N. (2018). Influence of Parental Healthy-Eating Attitudes and Nutritional Knowledge on Nutritional Adequacy and Diet Quality among Preschoolers: The SENDO Project. Nutrients.

[31] Haines J., Haycraft E., Lytle L., Nicklaus S., Kok F.J., Merdji M., Fisberg M., Moreno L.A., Goulet O., Hughes S.O. (2019). Nurturing Children’s Healthy Eating: Position statement. Appetite.

[32] Demir D., Bektas M. (2017). The effect of childrens’ eating behaviors and parental feeding style on childhood obesity. Eat. Behav..

[33] Mesas A.E., Muñoz-Pareja M., López-García E., Rodríguez-Artalejo F. (2012). Selected eating behaviours and excess body weight: A systematic review. Obes. Rev..

[34] Yee A.Z.H., Lwin M.O., Ho S.S. (2017). The influence of parental practices on child promotive and preventive food consumption behaviors: A systematic review and meta-analysis. Int. J. Behav. Nutr. Phys. Act..

[35] Lo K., Cheung C., Lee A., Tam W.W.S., Keung V. (2015). Associations Between Parental Feeding Styles and Childhood Eating Habits: A Survey of Hong Kong Pre-school Children. PLoS ONE.

[36] Mistry S.K., Puthussery S. (2015). Risk factors of overweight and obesity in childhood and adolescence in South Asian countries: A systematic review of the evidence. Public Health.

[37] Kołpa M., Jurkiewicz B., Norek J. (2017). Health Behaviour among Parents of Overweight and Obese Children. Health Promot. Phys. Act..

[38] Stąpor N., Kapczuk I., Krzewska A., Sieniawska J., Rakuś-Kwiatosz A., Piątek D., Bąk K., Szydełko J., Beń-Skowronek I. (2016). What is the difference in lifestyle in slim and overweight children?. Pediatr. Endocrinol..

[39] Zathey M., Wałęga A., Acedońska K., Balachowski J., Cieślak R., Drewnicka K., Halicka-Borucka M., Kasprzak M., Książek S., Kukuła M. (2021). Economic Analysis of Lower Silesia.

[40] (2013). World Medical Association Declaration of Helsinki: Ethical principles for medical research involving human subjects. JAMA J. Am. Med. Assoc..

[41] McIntosh W.A., Kubena K.S., Tolle G., Dean W.R., Jan J.S., Anding J. (2010). Mothers and meals. The effects of mothers’ meal planning and shopping motivations on children’s participation in family meals. Appetite.

[42] Landis J.R., Koch G.G. (1977). The measurement of observer agreement for categorical data. Biometrics.

[43] Colosi L. (2006). Designing an Effective Questionnaire.

[44] Acharya B. Questionnaire design. Proceedings of the Training-cum-Workshop.

[45] World Health Organization Reference Curves. https://ebook.ecog-obesity.eu/chapter-growth-charts-body-composition/world-health-organization-reference-curves/.

[46] Wądołowska L., Stasiewicz B., Gawęcki J. (2020). Procedura opracowania danych żywieniowych z kwestionariusza KomPAN^®^. KomPAN^®^ Kwestionariusz do Badania Poglądów i Zwyczajów Żywieniowych oraz Procedura Opracowania Danych.

[47] Tang D., Bu T., Dong X. (2020). Are parental dietary patterns associated with children’s overweight and obesity in China?. BMC Pediatr..

[48] Tenjin K., Sekine M., Yamada M., Tatsuse T. (2020). Relationship Between Parental Lifestyle and Dietary Habits of Children: A Cross-Sectional Study. J. Epidemiol..

[49] Di Cesare M., Sorić M., Bovet P., Miranda J.J., Bhutta Z., Stevens G.A., Laxmaiah A., Kengne A.P., Bentham J. (2019). The epidemiological burden of obesity in childhood: A worldwide epidemic requiring urgent action. BMC Med..

[50] Williams S.E., Greene J.L. (2018). Childhood Overweight and Obesity: Affecting Factors, Education and Intervention. J. Child. Obes..

[51] Raziani Y., Raziani S. (2020). Investigating the Predictors of Overweight and Obesity in Children. Int. J. Adv. Stud. Humanit. Soc. Sci..

[52] Adamo K.B., Brett K.E. (2014). Parental Perceptions and Childhood Dietary Quality. Matern. Child Health J..

[53] Kim H.S., Park J., Ma Y., Im M. (2019). What Are the Barriers at Home and School to Healthy Eating?: Overweight/Obese Child and Parent Perspectives. J. Nurs. Res..

[54] Newerli-Guz J., Kulwikowska K. (2014). Nutritional habits and preferences of pre-school children. Sci. J. Gdyn. Marit. Univ..

[55] Mushonga N.G.T., Mujuru H.A., Nyanga L.K., Nyagura S., Musaka N., Dembah R. (2017). Parental knowledge, attitudes and practices regarding overweight among preschool children in rural Zimbabwe. Afr. J. Food Agric. Nutr. Dev..

[56] Dallacker M., Hertwig R., Mata J. (2018). The frequency of family meals and nutritional health in children: A meta-analysis. Obes. Rev..

[57] Parikka S., Mäki P., Levälahti E., Lehtinen-Jacks S., Martelin T., Laatikainen T. (2015). Associations between parental BMI, socioeconomic factors, family structure and overweight in Finnish children: A path model approach. BMC Public Health.

[58] Bissinger A. (2020). Knowledge and Nutritional Behavior in Women between 25–35 Years of Age Depending on Education, Place of Residence and Family Situation. Master’s Thesis.

[59] Sharma H. (2022). How short or long should be a questionnaire for any research? Researchers dilemma in deciding the appropriate questionnaire length. Saudi J. Anaesth..

