# Supplementary material for: The Connection between Knowledge and the Nutritional Behaviour of Parents and the Occurrence of Overweight and Obesity among Preschool Children—A Pilot Study
Source: Nutrients. 2024 Jan 4;16(1):174. doi: 10.3390/nu16010174 (PMC10780658; doi:10.3390/nu16010174)
Supplement: Supplementary file 1 [file nutrients-16-00174-s001.zip › nutrients-2762425-supplementary.pdf]

S1.

CODE:

Questionnaire to investigate the knowledge and eating behaviour of preschool aged children's parents

I. Eating behaviour

1. How do you assess your diet?
*Please indicate **one** answer.*

- ☐ Very bad
- ☐ Bad
- ☐ Good
- ☐ Very good

2. Do you follow any diet?
*Please indicate **one** answer.*

- ☐ Yes*
- ☐ No

*If so, what kind of diet?: ……………………………………………………………………

3. How often do you eat in each place?
***One** answer per line should be given.*

	every day	several times per week	once per week	several times per month	once per month	several times per year	once per year	never
dairy bar								
canteen								
fast food restaurant								
restaurant								
home								

4. How often do you consume the following products?
***One** answer per line should be given.*

	several times per day	once per day	several times per week	once per week	1-3 times per month	never
vegetables (not applicable to potatoes, pulses)						
potatoes (not including fries or crisps)						
pulses (e.g. beans, soya, peas, chickpeas, etc.)						
fruits						

	several times per day	once per day	several times per week	once per week	1-3 times per month	never
whole-grain bread						
light bread (e.g. toast, rolls, croissants, etc.)						
white rice, light pasta, small groats (e.g. semolina, couscous, etc.)						
brown rice, coarse groats (e.g. buckwheat, barley, etc.), whole-grain pasta, oatmeal						
farinaceous products (e.g. dumplings, croissants, potato dumplings, pancakes, etc.).						
milk						
dairy products (e.g. yoghurt, kefir, buttermilk, etc.).						
yellow cheeses (including processed cheeses, moulded cheeses)						
curd cheeses (including homogenised cheeses, curd desserts), cottage cheeses						
cold cuts, sausages, wieners						
fish						
red meat (e.g. pork, beef, veal, etc.)						
white meat (e.g. chicken, turkey, etc.)						
butter						
margarine or mixes of butter with margarine						
lard						
instant products (e.g. powdered soups, ready-to-pour dishes, etc.)						
confectionery (e.g. sweets, biscuits, chocolate bars and other confectionery products)						
fast-food products (e.g. hamburgers, pizza, casseroles, etc.)						
salty snacks (e.g. sticks, crisps, etc.)						

5. How much water (e.g. mineral, table water) do you usually drink during the day?
*Please indicate **one** answer.*
- I don't drink water
- Up to 0.5 litres
- Approximately 1 litre
- From 1,5 to 2 litres
- Above 2 litres

6. How many meals do you usually eat during the day?
*Please indicate **one** answer.*
***Meal** – foods or combinations of foods customarily consumed at certain times of the day, e.g. morning, noon, evening.*
- 1 meal
- 2 meals
- 3 meals
- 4 meals
- 5 meals
- Above 5 meals

7. Do you eat meals at regular times during the day?
*Please indicate **one** answer.*
- No
- Yes, but only certain
- Yes, all of them

8. How often do you eat between meals (snack)?
*Please indicate **one** answer.*
***Snacking** – occasional consumption of products or sets of products between meals, mostly in small quantities.*
- Several times per day
- Once per day
- Several times per week
- Once per week
- 1-3 times per month
- Never

II. Non-food behaviour/physical activity

1. Does your child regularly attend organised physical activities (e.g. swimming, football training, dance, karate etc.) outside kindergarten?
*Please indicate **one** answer.*
- Yes*
- No

*If so, how many times per week?:…………………………………………………………

2. Does your child regularly do gymnastics at home?
*Please indicate **one** answer.*
- Yes, every day
- Yes, 5-6 times per week
- Yes, 3-4 times per week
- Yes, 1-2 times per week

- ☐ No

3. Does your child regularly spend at least one hour actively outdoors (e.g. cycling, playing in the playground, going for long walks)?
*Please indicate **one** answer.*
- ☐ Yes, every day
- ☐ Yes, 5-6 times per week
- ☐ Yes, 3-4 times per week
- ☐ Yes, 1-2 times per week
- ☐ No

III. Nutritional knowledge

1. How would you rate your nutritional knowledge?
*Please indicate **one** answer.*
- ☐ Insufficient
- ☐ Sufficient
- ☐ Good
- ☐ Very good
- ☐ I don't know

2. In your opinion, is proper nutrition of preschool children a prerequisite for their optimal physical, mental and social development?
*Please indicate **one** answer.*
- ☐ Yes
- ☐ No
- ☐ I don't know

3. In your opinion, how many meals during a day should a child eat?
*Please indicate **one** answer.*
- ☐ 2 meals
- ☐ 3 meals
- ☐ 4 meals
- ☐ 5 meals
- ☐ I don't know

4. What proportion of the daily energy needs should be covered by meals consumed in a kindergarten?
*Please indicate **one** answer.*
- ☐ 30%
- ☐ 50%
- ☐ 75%
- ☐ 90%
- ☐ I don't know

5. From which source do you think the largest percentage of energy should come in a child's diet?
*Please indicate **one** answer.*
- ☐ Simple carbohydrates (e.g. confectionery, fruit)
- ☐ Complex carbohydrates (e.g. vegetables, whole grains)
- ☐ Animal fats (e.g. butter, lard)

- Plant-based oils (e.g. avocados, nuts and seeds, plant oils)
- Animal protein (e.g. meat, fish, milk, yoghurt)
- Plant protein (e.g. pulses, plant-based drinks/yoghurts - soya, coconut, oat, etc.)
- I don’t know

6. In your opinion, what is a universal drink with which children should quench their thirst?
*Please indicate **one** answer.*

- Juice
- Sweetened beverage of the "cola" type
- Water
- Tea
- Coffee
- Milk
- Flavoured water
- I don’t know
- Other (what kind of?):..

7. How many portions of milk and/or dairy products do you think a child should consume during a day?
*Please indicate **one** answer.*
***1 portion of milk, dairy products** – 1 glass of milk, yoghurt, kefir or buttermilk, 100 grams of cottage cheese, 2 slices of yellow cheese, packet of homogenised cottage cheese, granulated cottage cheese.*

- 1-2 portions
- 3-4 portions
- 5-6 portions
- Above 6 portions
- I don’t know

8. In your opinion, should a preschool child eat pulses (e.g. beans, broad beans, chickpeas, lentils, etc.)?
*Please indicate **one** answer.*

- Yes
- No
- I don’t know

9. In how many meals per day do you think fruits and vegetables should be included in your child's diet?
*Please indicate **one** answer.*

- In one meal
- In two meals
- In three meals
- In all meals
- I don’t know

10. Which articles should predominate in a child's diet - vegetables or fruits?
*Please indicate **one** answer.*

- Vegetables
- Fruits
- I don’t know

11. In your opinion, what meat should be in children's diets most often?
*Please indicate **one** answer.*
- ☐ Poultry
- ☐ Veal
- ☐ Beef
- ☐ Pork
- ☐ I don't know

12. What is the maximum amount of salt that can be in a child's diet per day?
*Please indicate **one** answer.*
- ☐ 1 teaspoon
- ☐ 2 teaspoons
- ☐ 3 teaspoons
- ☐ There is no correct answer
- ☐ I don't know

13. Would you like the idea of the organisation, by the kindergarten your child attends, classes connected with proper child's nutrition?
*Please indicate **one** answer.*
- ☐ Yes
- ☐ No

IV. Metric

Please complete your details (only questions 1st and 2nd respectively relate to your child's age and gender).

1. Child's age (in years): ....

2. Child's gender:
- ☐ Girl
- ☐ Boy

3. Parent's/ legal guardian's age (in years): ....

4. Parent's/ legal guardian's gender:
- ☐ Woman
- ☐ Man

5. Place of residence:
- ☐ Village
- ☐ City of up to 100 000 inhabitants
- ☐ City with more than 100 000 inhabitants

6. Education:
- ☐ Primary
- ☐ Secondary
- ☐ Professional
- ☐ Higher

7. Professional status:
- ☐ Student
- ☐ Working
- ☐ Unemployed
- ☐ On a pension
- ☐ Other: ....

8. Parent's/legal guardian's body weight (in kilograms): ....

9. Parent's/legal guardian's body height (in centimetres):..
